# Supplementary material for: A repressor-decay timer for robust temporal patterning in embryonic Drosophila neuroblast lineages
Source: eLife. 2018 Dec 10;7:e38631. doi: 10.7554/eLife.38631 (PMC6303102; doi:10.7554/eLife.38631)
Supplement: Supplementary file 1. [file elife-38631-supp1.docx]

**Supplementary File 1**

# Model equations and Parameters

The molecular interactions driving the embryonic NB timer have been well described (Grosskortenhaus et al., 2005; Grosskortenhaus et al., 2006; Isshiki et al., 2001; Tran and Doe, 2008). These interactions appear to integrate the two core timers discussed in the main text: some genetic interactions are compatible with the decay-based timer, while other interactions are compatible with relay-based timer. Based on this existing knowledge, we formulated a mathematical model capturing the described interactions. This model allows for variable influence of both timer types of based on parameter choice. The following ODEs describe our TTF timer model:

$$(7) \frac{d[hb]}{dt}= \theta\left( t-t_{0} \right)\beta_{hb}-\alpha_{hb}[hb]$$

$$\left( 8 \right)\frac{d[kr]}{dt}=\beta_{kr}H_{A}\left( [hb] \right)H_{R}\left( [pdm] \right)+\theta\left( t-t_{0} \right)\beta_{kr}^{basal}H_{R}\left( [pdm] \right)-\alpha_{kr}[kr]$$

$$\left( 9 \right)\frac{d[pdm]}{dt}= \beta_{pdm}H_{A}\left( [kr] \right)H_{R}\left( [hb] \right)H_{R}\left( [cas] \right)+\beta_{pdm}^{basal}H_{R}\left( [hb] \right)H_{R}\left( [cas] \right)-\alpha_{pdm}[pdm]$$

$$\left( 10 \right)\frac{d[cas]}{dt}= \beta_{cas}H_{A}\left( [pdm] \right)H_{R}\left( [hb] \right)H_{R}\left( [kr] \right)+\beta_{cas}^{basal}H_{R}\left( [hb] \right)H_{R}\left( [kr] \right)-\alpha_{cas}[cas]$$

Notations and additional parameters:

$\theta\left( t-t_{0} \right)$ it a temporal step function which allows the production of hb only at t<t_0_.

For the TTF i, $\beta_{i}$ is i’s production rate, $\alpha_{i}$ is i’s degradation rate, $\beta_{i}^{basal}$ is i’s basal production rate in the absence of any activators.

$H_{A}\left( [i] \right)=\frac{1}{1+{(\frac{K_{i,j}}{[i]})}^{n_{i}}}$ , is a hill function representing transcriptional activation by the TTF i of gene j. With $K_{i,j}$, the K_D_ for i activity on j, and $n_{i}$ , i’s hill coefficient.

$H_{R}\left( [i] \right)=\frac{1}{1+{(\frac{[i]}{K_{i,j}})}^{n_{i}}}$ , is a hill function representing transcriptional repression by the TTF i. With $\boldsymbol{K}_{\boldsymbol{i}}$, the K_D_ for i activity, and $\boldsymbol{n}_{\boldsymbol{i}}$ , i’s hill coefficient. Additional model parameters are the $\boldsymbol{Tr}_{\boldsymbol{i}}$, which are the thresholds. Only when the concentration of i, [i], is above $\boldsymbol{Tr}_{\boldsymbol{i}}$, i is considered to be “on”. $\mathbf{t}_{\mathbf{end}}$ is the duration of the simulation. We solve this full set of ODEs numerically using a standard MATLAB ODE solver. The model equations insure the dominance of the repressors: a gene will not be expressed in the presence of its repressor even if an activator is also present. This property which stems from the multiplication of the production rates by the $\boldsymbol{H}_{\boldsymbol{A}}\left( \boldsymbol{[i]} \right)\boldsymbol{H}_{\boldsymbol{R}}\left( \boldsymbol{[i]} \right)$ term was observed experimentally (Grosskortenhaus et al., 2005; Grosskortenhaus et al., 2006; Isshiki et al., 2001; Nakajima et al., 2010; Tran and Doe, 2008; Tran et al., 2010).

In this model, the relative significance of specific interactions in determining the temporal dynamics, depends on the choice of parameters. Each interaction can be indirectly “strengthened” or “weakened” by appropriate parameter selection. For example, let us consider the up regulation of Pdm and the relative importance of repression of by Hb and activation by Kr for this process. In the WT, these interactions are described by the first term in equation 9: $\beta_{pdm}H_{A}\left( [kr] \right)H_{R}\left( [hb] \right)H_{R}\left( [cas] \right)+\beta_{pdm}^{basal}H_{R}\left( [hb] \right)H_{R}\left( [cas] \right)$. When discussing Pdm **induction**, we can disregard the term $H_{R}\left( [cas] \right)$, which represents repression of Pdm by Cas since Cas is induced later than Pdm in the cascade. We therefore focus on the balance between Kr-dependent transcription $\beta_{pdm}H_{A}\left( [kr] \right)H_{R}\left( [hb] \right)$ and basal transcription independent from Kr: $\beta_{pdm}^{basal}H_{R}\left( [hb] \right)$. The relative magnitude of the production rates $\beta_{pdm}$ and $\beta_{pdm}^{basal}$ , together with other parameters, will determine how important is activation by Kr for timely Pdm induction. For example: when $\beta_{pdm}^{basal}$ >>$\beta_{pdm}$, induction by Kr will be negligible and downregulation of Hb would be dominant. For $\beta_{pdm}^{basal}$ <<$\beta_{pdm}$, the opposite is true: Kr-dependent transcription of Pdm is much more substantial.

# References

Grosskortenhaus, R., Pearson, B.J., Marusich, A., Doe, C.Q., 2005. Regulation of temporal identity transitions in Drosophila neuroblasts. Dev Cell 8, 193-202.

Grosskortenhaus, R., Robinson, K.J., Doe, C.Q., 2006. Pdm and Castor specify late-born motor neuron identity in the NB7-1 lineage. Genes Dev 20, 2618-2627.

Isshiki, T., Pearson, B., Holbrook, S., Doe, C.Q., 2001. Drosophila neuroblasts sequentially express transcription factors which specify the temporal identity of their neuronal progeny. Cell 106, 511-521.

Nakajima, A., Isshiki, T., Kaneko, K., Ishihara, S., 2010. Robustness under functional constraint: the genetic network for temporal expression in Drosophila neurogenesis. PLoS Comput Biol 6, e1000760.

Tran, K.D., Doe, C.Q., 2008. Pdm and Castor close successive temporal identity windows in the NB3-1 lineage. Development 135, 3491-3499.

Tran, K.D., Miller, M.R., Doe, C.Q., 2010. Recombineering Hunchback identifies two conserved domains required to maintain neuroblast competence and specify early-born neuronal identity. Development 137, 1421-1430.
