# Supplementary material for: A repressor-decay timer for robust temporal patterning in embryonic Drosophila neuroblast lineages
Source: eLife. 2018 Dec 10;7:e38631. doi: 10.7554/eLife.38631 (PMC6303102; doi:10.7554/eLife.38631)
Supplement: Supplementary file 2. — Neuronal fates for co-expression of TTFs in the NB at time of division were deduced from lineages described in Figure 1D. For every combination, the resulting fate is specified along with the genotypes from Figure 1D from which fate was deduced. Constitutive expression genotypes are denoted by const and deletions by Δ. [file elife-38631-supp2.docx]

| TTF combination | Neuronal fate | Reference genotype |
| --- | --- | --- |
| Hb | 1,2 | WT, const Hb, ΔHb |
| Hb,Kr | 1,2 | WT, const Hb, ΔHb |
| Kr | 3,Unknown | 3:WT, ΔHb, Unknown :this study (Fig.4E) |
| Kr,Pdm | 3 | WT,const Kr |
| Pdm | 4 | WT, ΔPdm |
| Pdm,Cas | 5,IN | 5:WT, ΔPdm, ΔCas,const Pdm, , IN :this study (Fig.4E) |
| Cas | IN | const Cas |
| Hb,Pdm | 1,2 | const Pdm |
| Kr,Cas | 3 | const Cas |
