# Supplementary material for: A repressor-decay timer for robust temporal patterning in embryonic Drosophila neuroblast lineages
Source: eLife. 2018 Dec 10;7:e38631. doi: 10.7554/eLife.38631 (PMC6303102; doi:10.7554/eLife.38631)
Supplement: Supplementary file 3. [file elife-38631-supp3.docx]

| Figure, panel | Number of NB7-1s per box left to right |
| --- | --- |
| 4,C | 105,100 ,48, 81, 103, 90, 70,70,134,116, 63,77 |
| 4,E | 9,19,27,31,28,20,7,3 |
| 5,A | 105,100, 129 ,263 ,320 ,140 |
| 5,B | 31,28,22,26,22,16 |
| 5,C | 20,19,40 ,81 ,34 ,34 |
| 5,D | 19,35,18,38,28,35 |
| 5,E | 9,39,36,36,47,55 |
| 5,F | 17,27,27,40,27,44 |
