# Supplementary material for: A repressor-decay timer for robust temporal patterning in embryonic Drosophila neuroblast lineages
Source: eLife. 2018 Dec 10;7:e38631. doi: 10.7554/eLife.38631 (PMC6303102; doi:10.7554/eLife.38631)
Supplement: Supplementary file 4. — Drawing was done from a log-uniform distribution on indicated ranges. When no specific TTF is indicated for the parameter, it is the same for all four TTFs. [file elife-38631-supp4.docx]

| Parameter | Units | Range or values |
| --- | --- | --- |
| $t_{0}$ | $t$[min] | 20 or 50 |
| $t_{end}$ | $t$[min] | 280 |
| $\beta_{i}$ | $t^{-1}C$ | [1, 3000] |
| $\beta_{kr}^{basal}$ | $t^{-1}C$ | 0 for WT and all perturbations except Hb deletion. [1, 1000] for Hb deletion |
| $\beta_{i}^{basal}, i\in\{Pdm,Cas\}$ | $t^{-1}C$ | [1,800] |
| $\alpha_{i}$ | $t^{-1}$ [1/min] | [10^-3,1] |
| $K_{i,j}$ | $C^{n_{w}}$ | [0.01,100] |
| ${Tr}_{i}$ | C | 2 |
| $n_{i}$ | - | 5 |
